# *Txnip* expression promotes JNK-mediated neuronal death in response to reactive oxygen species

**DOI:** 10.3389/fnmol.2023.1210962

**Published:** 2023-07-20

**Authors:** Brenda García-Hernández, Julio Morán

**Affiliations:** División de Neurociencias, Instituto de Fisiología Celular, Universidad Nacional Autónoma de México, Mexico City, Mexico

**Keywords:** Thioredoxin Interacting Protein, FOXO3, reactive oxygen species, apoptosis, cerebellar granule neurons, MAPK, Akt

## Abstract

TXNIP is a protein sensitive to oxidant conditions whose expression is related to the progression of death in cancer, diabetes, ischemia, and neurodegenerative diseases, among others. Because of this, many studies propose TXNIP as a therapeutic target in several diseases. Exposure of cerebellar granule neurons to staurosporine or low potassium leads to apoptotic death. Both conditions generate an early production of reactive oxygen species (ROS) that induces the activation of the ASK1 pathway and the apoptotic machinery. In these models, it has been shown an increase in TXNIP protein mediated by ROS. Here, we evaluated the molecular mechanisms involved in the regulation of the *Txnip* expression during neuronal death, as well as the role of the protein in the progression of cell death induced by these two apoptotic conditions. In cultured cerebellar granule neurons, we observed that low potassium and staurosporine induced an early increase in ROS that correlated with an increase in *Txnip* mRNA. When we evaluated the promoter of the gene, we found that the JASPAR-reported FOXO1/3 transcription factor motifs are close to the transcription start site (TSS). We then verified through the Chromatin immunoprecipitation technique (ChIP) that FOXO3 interacts with the *Txnip* promoter after 1 h of low potassium treatment. We also detected FOXO3 nuclear translocation by low potassium and staurosporine treatments. Finally, by using shRNA in the neuroblastoma MSN cell line, we found that *Txnip* downregulation decreased neuronal death induced by staurosporine stimulus. Together, these results suggest that ROS promotes the expression of *Txnip* through the activation of the FOXO3 transcription factor mediated by Akt inhibition. We also demonstrated that TXNIP is necessary for neuronal death progression.

## 1. Introduction

Thioredoxin Interacting Protein (TXNIP) is a member of the arresting protein family that presents two arrestin-like domains and four splicing variants in humans (Tsubaki et al., 2020). The gene sequence in humans, mice, and rats is highly conserved. The protein in humans and mice shares close sequence homology (96%) suggesting its relevance in cell signaling (Singh, 2013; Zhou and Chng, 2013). TXNIP is considered a death inductor in pancreatic beta cells and its expression levels correlate with the grade, stage, and metastasis capacity in breast cancer, hepatocellular cancer, bladder cancer, and leukemia (Zhou et al., 2010; Yoshihara et al., 2013; Zhou and Chng, 2013). For this reason, it is recognized as a tumor suppressor molecule (Zhou and Chng, 2013). TXNIP can bind thioredoxin (Trx) forming a complex that is called redoxisome, which participates in modulating different processes by sensing the redox environment. In previous studies, it was found that TXNIP expression is markedly increased in cerebellar granule neurons (CGN) in response to apoptotic conditions induced by low potassium (K5) (Saitoh et al., 2001; Zaragoza-Campillo and Morán, 2017) or staurosporine (Sts) (Zaragoza-Campillo and Morán, 2017). Furthermore, we have also previously found that TXNIP protein induction is dependent on reactive oxygen species (ROS) production in this model (Zaragoza-Campillo and Morán, 2017).

Reactive oxygen species (ROS) are molecules resulting from the sequential reduction of molecular oxygen (Lambeth, 2004; Covarrubias et al., 2008; Olguín-Albuerne and Morán, 2018). When ROS levels increase above physiological concentrations, generalized cell damage and death occur. This condition is known as oxidative stress. However, under low concentrations, ROS may participate as signaling molecules through the specific and reversible oxidations of several proteins depending on cell type and the cellular context, among others. In this regard, ROS may induce post-translational modifications and modulate several cellular events, including proliferation, differentiation, and cell death (Finkel, 2011; Olguín-Albuerne and Morán, 2018).

In previous studies, we observed an early increase in ROS production during apoptotic death of NGC induced by K5 and Sts (Valencia and Morán, 2001; Cid-Castro and Morán, 2021). In this early phase of apoptotic death, we also observed an Akt inactivation in both models, which was dependent on ROS increase (Zaragoza-Campillo and Morán, 2017). On the other hand, we demonstrated that apoptotic death induced by K5 and Sts also activated the mitogen-activated protein kinases (MAPK) pathway during the first hours of the process, particularly JNK and p38, whose pharmacological inhibition markedly reduced neuronal death (Ramiro-Cortés and Morán, 2009; Ramiro-Cortés et al., 2011).

There is not enough information about the role of TXNIP in neuronal death and whether this molecule could be related to the activation of the MAPK pathway during cell death. It is possible that TXNIP could recruit Trx from ASK1, which would lead to the activation of the MAPK pathway and, consequently, to neuronal death. ASK1 is a member of the MAPK family, which is inactive when bound to Trx. When Trx dissociates from this complex, an active form of ASK1 is produced and then the JNK and p38 signaling pathways are activated (Yoshihara et al., 2013). Together, these cascades of events ultimately lead to the execution of the cell death program. Thus, the ROS-TXNIP-Trx-ASK1-JNK/p38 cascade may play a critical role in the activation of apoptosis (Shen and Pervaiz, 2006; Zhou and Chng, 2013).

It has been established that *Txnip* promoters share common motifs for different transcription factors such as CREB, NFKB, HIF1 alpha, GR, and FOXOs (Wang et al., 2006; Baker et al., 2008; Tsubaki et al., 2020) and direct evidence in neurons shows a possible involvement of the FOXO family of transcription factors in cell development, apoptosis (Papadia et al., 2008; Webb et al., 2016), oxidative stress, and autophagy transcriptional responses (van der Heide et al., 2004; Tzivion and Hay, 2011; Hemmings and Restuccia, 2012). In neurons, FOXO1 and FOXO3 could be responsible for *Txnip* up-regulation (Papadia et al., 2008; Webb et al., 2016). In addition, FOXOs can be regulated by Akt through the phosphorylation of specific amino acid residues. FOXO3 presents Akt motifs in Thr 32, Ser 253, and Ser 315 and FOXO1 in Ser 256, Thr 24, and Ser 319. Phosphorylated FOXOs are chaperoned by 14–3-3 protein, hiding the FOXO-DNA binding sites, recruiting it in the cytoplasm, and inhibiting its function. Therefore, Akt inactivation is crucial for FOXO transcriptional activity through this pathway. Non-phosphorylated FOXO translocates to the nucleus and induces the expression of target genes (Taylor et al., 2015; Zhang et al., 2019).

As mentioned above, there is not much information on the transcriptional regulation of *Txnip* and the involvement of the protein in CGN death initiation/progression. Thus, the aim of this work is to describe the regulation of its expression during neuronal death induced by K5 and Sts, evaluate the mechanisms of FOXO transcription factors in *Txnip* regulation, and explore the role of TXNIP in neuronal death.

## 2. Materials and methods

Fetal calf serum, penicillin/streptomycin, basal Eagle’s- and RPMI- medium were from GIBCO (Grand Island, NY, United States). Dihydroethidium (DHEt) and calcein-AM were purchased from Molecular Probes (Eugene, OR, United States). Trizol reagent, M-MVL reverse transcriptase and Oligo (dT) were from Invitrogen (Carlsbad, CA, United States). Poly-l-lysine (mol. wt. > 300,000), trypsin, trypsin inhibitor, DNAse, cytosine arabinoside, DMSO, staurosporine, and LY294002 were acquired from Sigma (St. Louis, MO, United States). Peroxidase-conjugated anti-mouse was purchased from Jackson Immuno Research (West Grove, PA, United States). Protease inhibitor cocktail tablets (Complete) were purchased from Roche (Mannheim, Germany). TXNIP rabbit antibody (D5F3E) and Alexa 488 antibody (4412S) were acquired from Cell Signaling Technology (Danvers, MA, United States). Vechtashield mounting medium with DAPI was from Vector Laboratories (Burlingame, CA, United States). shRNA *Txnip* (RSH045604) was purchased from GeneCopoeia, and Oligo DT and DNTPs were from Thermo Fisher Scientific (Carlsbad, CA, United States). MasterMix qPCR ROx PyroTaq EvaGreen 5x was from Biotium. Hydrogen peroxide, Dynabeads. FOXO1 (ab39670), FOXO3A (ab12162), and ChIP-grade antibodies were purchased from Abcam. ChIP primers were purchased from Sigma (St.Louis, MO, United States) and FOXO3 antibody for immunofluorescence was acquired from Cell Signaling (Danvers, MA, United States). JNK inhibitor SP600125 was purchased from Tocris (Ellisville, MO, United States). Reverse transcriptase was acquired from InvitrogenTM (Carlsbad, CA, United States). ProSiev QuadColor protein marker and Nucleofector VPI-1003 were from Lonza (Basel, Switzerland).

### 2.1. Cell culture

All animals used for the experimentation were treated in accordance with the accepted standards of animal care and with the procedures approved by the local Committee of Research and Ethics of the Instituto de Fisiología Celular, Universidad Nacional Autónoma de Mexico (protocol number: JMA175-21). The protocol used followed the Guidelines for the Care and Use of Mammals in Neuroscience as well as guidelines released by the Mexican Institutes of Health Research and the National Institutes of Health guide for the care and use of Laboratory animals. All efforts were made to minimize animal suffering and to reduce the number of animals used.

Cerebellar granule neurons (CGN) cultures were prepared as previously described (Moran and Patel, 1989). CGN were obtained from 8 -day- old Wistar rats or from 4-day-olds in ICR mice cerebellum and plated at a 265 × 10^3^ cell/cm^2^ density in plastic dishes coated previously with poly-l-lysine (5 μg/mL). The culture medium contained basal Eagle’s medium supplemented with 10% (v/v) heat-inactivated fetal calf serum, 2 mM glutamine, 25 mM KCl, 50 μg/mL streptomycin, and 50 U/mL penicillin. Cytosine arabinoside (10 𝜇M) was added 24 h after seeding to prevent the proliferation of nonneuronal cells. Cultures were maintained under these conditions for 8 days and kept at 37°C in an atmosphere of CO^2^ (5%) and saturated air with water vapor (95%). At the end of the preparation, CGN cultures contained approximately 95% neurons. Apoptotic death of CGN was induced by two different stimuli: serum-free medium containing 5 mM KCl (K5) or by 0.5 𝜇M staurosporine (Sts) administration. In the CGN viability assays with short exposure to K5 and Sts we used K25 conditioned medium to prevent neuronal death by fresh medium, as has been observed.

A human MSN neuroblastoma cell line was donated by Dr. Clorinda Arias from the Institute of Biomedical Science of the National Autonomous University of Mexico. The cell line was cultured in accordance with Ferrera et al. (2017). Adherent MSN cells were seeded in a monolayer with RPMI 160 medium supplemented with 10% of fetal bovine serum (Gibco, Life Technologies Corporation, Grand Island, NY, United States), 1% of non-essential MEM, and 1% glutamine-serine-asparagine. The cells were maintained in humified incubation with 95% of air and 5% of CO^2^ and 37°C. The cells were seeded in a density of 1 × 10^6^ cells /100 mm dish (Reynolds et al., 1986).

### 2.2. Reactive oxygen species quantification

ROS levels were quantified by dihydroethidium. DHE is a fluorogenic probe for the detection of intracellular superoxide that is oxidized to ethidium and intercalates into nucleic acids emitting fluorescence at 610 nm (Cho and Hwang, 2011). DHE is irreversibly oxidized and therefore the signal obtained is the result of the sum of the previous accumulation. CGN were seeded in a multi-well plate and treated with 10 μM dihydroethidium (DHEt) for 30 min at 37°C. Cells were observed in an epifluorescence microscope with a rhodamine filter with a wavelength of 488–515 nm. DHEt diffuses into cells and is oxidized by ROS in the cytosol producing ethidium and 2- hydroxy ethidium that binds to the DNA and emits bright red fluorescence. Fluorescence images were acquired, and fluorescence intensity was measured with the Image J Program. The data were normalized with respect to K25 conditions.

### 2.3. Cell viability

Cell viability was measured by calcein-AM accumulation and propidium iodide (PI) exclusion to stain live and dead cells, respectively. Endogenous esterases in living cells transform calcein-AM to calcein, a green-fluorescent product. The PI crosses the plasma membrane of damaged cells; it binds to DNA and double-stranded RNA by intercalation and emits an intense red fluorescence signal. This dye shows weak fluorescence when it is not bound and in an aqueous solution. Cells were incubated with calcein-AM (1 *𝜇*M) and PI (40 𝜇M) for 30 min at 37°C and were observed in an epifluorescence microscope. Stained cells were counted with Image J software.

### 2.4. RT-qPCR

Cerebellar Granule Neurons cultured for 7 days were used after treatments with K5, staurosporine, LY4002902 (30–100 μΜ), or hydrogen peroxide 100 μM. After harvesting the cells, total RNA was purified using TRIzol reagent and 0.5 μΜ of total RNA was reverse transcribed to cDNA by using Reverse aid Reverse Transcriptase (Thermo Scientific) and Oligo DT. A measure of 50 ng cDNA was used for qPCR and the MasterMix qPCR ROx PyroTaq EvaGreen 5x. Primer sequences were used at 10 μM. TXNIP forward 5’ GGCTGGGGGTGTTGTTTA 3’ Reverse 5’ GCGCAGGAAAATAAGATGA GAPDH Forward 5’CTCATGAC CACAGTCCATGC 3’ Reverse 5’TTCAGCTCTGGGATGACCTT 3′. PCR assays were performed in three replicates in each group. All results were analyzed by the Delta Ct method.

### 2.5. Promoter analysis

Promoter analysis was performed by using the NCBI database for the Txnip gene sequence. Two motifs reported by the JASPAR database for FOXO1 and FOXO3 in mice were searched. We analyzed the promoter by using NCBI upstream sequence for the *Txnip* gene in mice (accession number NM_023719). First, we localized the E-box sequences in the promoter and determined the transcription start site (TSS) using JASPAR-reported FOXO motifs in mice^1^ and we found two motifs close to the TSS -1,000 nt upstream. The first is located at -560 nt and is specific for FOXO1 and we named it motif 1; the second, at -260 nt is common for FOXO1 and FOXO3 and we named it motif 2. We then analyzed promoter sequences in humans and rats (NCBI accession number NM_006472 and NM_001008767) and we found that these motifs are conserved across these species.

### 2.6. Chromatin immunoprecipitation FOXO 3 in *Txnip* promoter

We used 1 × 10^7^ neurons of 7 DIV for each experimental condition: K25, K5, for 30 and 60 min and 100 μM hydrogen peroxide for 1 h. Cells were crosslinked with 1.1% formaldehyde for 10 min. The formaldehyde linking was quenched by adding 125 mM glycine for 10 min. Cells were pelleted and rinsed once in cold phosphate-buffered saline (PBS). Nuclear pellets were obtained by using RIPA buffer (50 mM Tris HCl pH 8.0, 150 mM NaCl, 1% NP-40, 0.5% deoxycholate 0.1% SDS, and Complete protease inhibitor cocktail (Roche)) and sonicated with a sonicator (Branson Analog) set at 400 w power and ½ mm point out the power of 10% with 8 cycles of 15 s ON/30 s OFF. Sonicated chromatin was centrifugated at 13,000 rpm for 15 min. A binding buffer was used (10 mM Tris–HCl, pH 8.0, 300 mM NaCl, 5 mM EDTA, pH 8.0, 0.5% SDS). Chromatin was incubated overnight at 4°C with 5 μg/sample of FOXO 1 polyclonal anti-rabbit (ab39670) and FOXO 3 polyclonal anti-rabbit ab ab12162 or with TFII ab as a control. The antibody was incubated overnight at 4°C in rotation. Dynabeads (Thermo Scientific) was used to immunoprecipitate. The beads were washed three times with binding buffer, precipitated on the magnetic strip, and resuspended in the same volume with binding buffer. The beads were washed with the following buffers: low salt buffer (50 mM Tris–HCl, pH 8.1, 150 mM NaCl, 1 mM EDTA, 0.1% SDS, 1% Triton X-100, 0.1% sodium deoxycholate), high salt buffer (50 mM Tris–HCl, pH 8.1, 500 mM NaCl, 1 mM EDTA, 0.1% SDS, 1% triton X-100, 0.1% sodium deoxycholate), LiCl salt buffer (50 mM Tris–HCl, pH 8.1, 1 mM EDTA, pH 8.0, 1% NP-40, 0.7% sodium deoxycholate, 500 mM LiCl_2_) and TE buffer (10 mM Tris–HCl, pH 8.0, 1 mM EDTA, pH 8.0). All samples had16 μl of 5 M NaCl added. We incubated samples overnight at 65°C with shaking to remove protein-DNA interaction. DNA was purified with phenol: chloroform including the input samples. PCR was performed as described above. Motif 1 FOXO1 forward 5’ GGCTGGGGGTGTTGTTTA 3’ Reverse 5’GCGCAGGAAAATA AAGATGA 3’ Motif 2 FOXO’s forward 5’ TAAAACAAGGGCCA AGTAGCC 3’ Reverse 5’ ATATAGCCGCCTGGCTTG 3’.

### 2.7. Immunofluorescence

Anti-FOXO3 cell signaling antibody (D19A7) and secondary antibody with Alexa Fluor 488 anti-rabbit cell signaling (4412S) were used. Cells were grown on glass coverslips in a 12-well plate over a 5 x PLL layer, maintained in K25 medium for 7DIV, and treated with the PI3K inhibitor LY294002 (100 μM for 4 h) were treated with K5 medium or Sts (0.5 μM) for 30, 60 or 120 min or treated with 100 μM hydrogen peroxide for 1 h. Subsequently, the cells were washed three times with PBS, fixed with 4% paraformaldehyde for 20 min at room temperature, washed three times with PBS, and blocked with 3% serum-0.2% triton-PBS for 2 h at room temperature. The primary antibody was incubated (1:500) in 3% goat serum PBS-T solution overnight. The secondary antibody coupled to Alexa 488 (1:1000) was incubated for 1 h at room temperature in the dark. Vechtashied mounting medium with DAPI was used. The images were captured in a Zeiss LSM800 confocal microscope and images were taken in Z Pearson’s colocalization coefficient was measured by using the JACoP plugin for Image J.

### 2.8. Cell electroporation

The MSN neuroblastoma cells were transfected before plating with shRNA TXNIP using the nucleofection electroporation technique with the Amaxa VP1003 kit using the C-13 program. GFP reporter-TXNIP shRNA and a scramble control sequence (Scr) were transfected. For every 4 million cells, 5 μg of the Maxiprep-purified plasmid was used. Cells were plated in 35 mm Costar dishes. The transfection efficiency was evaluated at 48 h with an epifluorescence microscope as the total number of GFP-positive cells relative to the total number of cells. The transfection efficiency was 28% for both scramble and shRNA transfected cells.

### 2.9. Western blot

Neuroblastoma MSN cells were cultured and transfected as previously described and after 48 h cells were washed with cool PBS buffer and homogenized with RIPA lysis buffer (25 mM Trizma, 50 mM NaCl, 2% Igepal, 0.2% SDS, pH 7.4) with complete protease inhibitor (Roche), phosphatase inhibitor (Roche) and 50 mM NaF. Cells were sonicated for 30 s with 30% of sonication potency and centrifuged at 4,5000 rpm for 5 min. Protein content was determined by Lowry assay. Electrophoresis was performed on 10% SDS-PAGE with 20 μg per well. Gels were transferred onto PVDF membranes at 300 mA for 1 h. Membranes were blocked with TTBS buffer (100 mM Tri-HCl, 150 mM NaCl and 0.1% Tween, pH 7.4) with 5% fat-free milk for 1 h under shaking at room temperature. Cell signaling TXNIP rabbit antibody (D5F3E) (1:1000) and peroxidase-conjugated anti-rabbit (1:10,000) were used.

### 2.10. Statistical analysis

Data are presented as the mean ± the standard error of the mean. The Prism Graph 8.1 program was used to perform the statistical analysis of the experiments. The normal distribution of the data was determined with a Shapiro-Wilks test. Viability experiments are presented as a percentage normalized to K25. PCR data are normalized to the GAPDH levels and the K25 control relative to 1 and ChIP data were normalized to the background. Statistical significance was determined as *p* < 0.05. *p* values are represented as NEJM style *p < 0.033, **p < 0.002, ***p < 0.001. Data were analyzed by using the ANOVA test followed by Dunnett’s *post hoc* test. For multivariable data such as ChIP and TXNIP shRNA viability assay, a two-way ANOVA followed by Tukey’s test or Sidak’s test was used.

## 3. Results

### 3.1. Staurosporine and low potassium induce neuronal death

**Figure 1:**
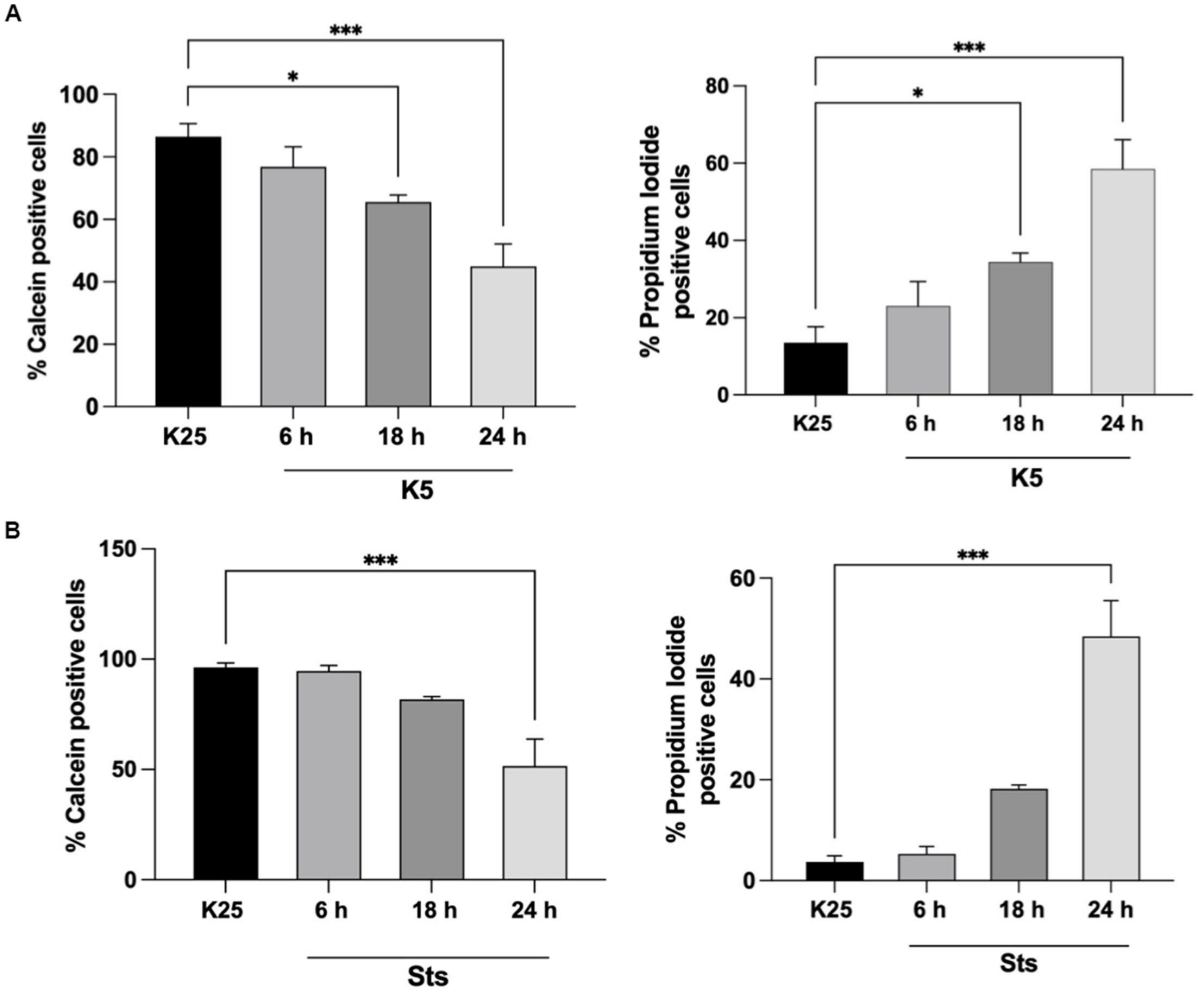
Cell viability decreases after 18-24 h of K5 or Sts treatment. Seven DIV CGN were switched to a low potassium medium (K5) **(A)** or treated with Sts (0.5 mM) **(B)** for 6, 18, 24 h, and neuronal viability was measured by using Calcein and propidium iodide (PI) dyes as detailed in Methods. Cell viability is expressed as the percentage of Calcein (living cells) or PI (dead cells) positive cells from total cells (Calcein plus PI-positive cells). Bars represent the means ± SEM of 5 independent experiments, *p < 0.033, ***p < 0.001. Data were analyzed with one-way ANOVA followed by Dunnett’s multiple comparison test.

**Figure 2:**
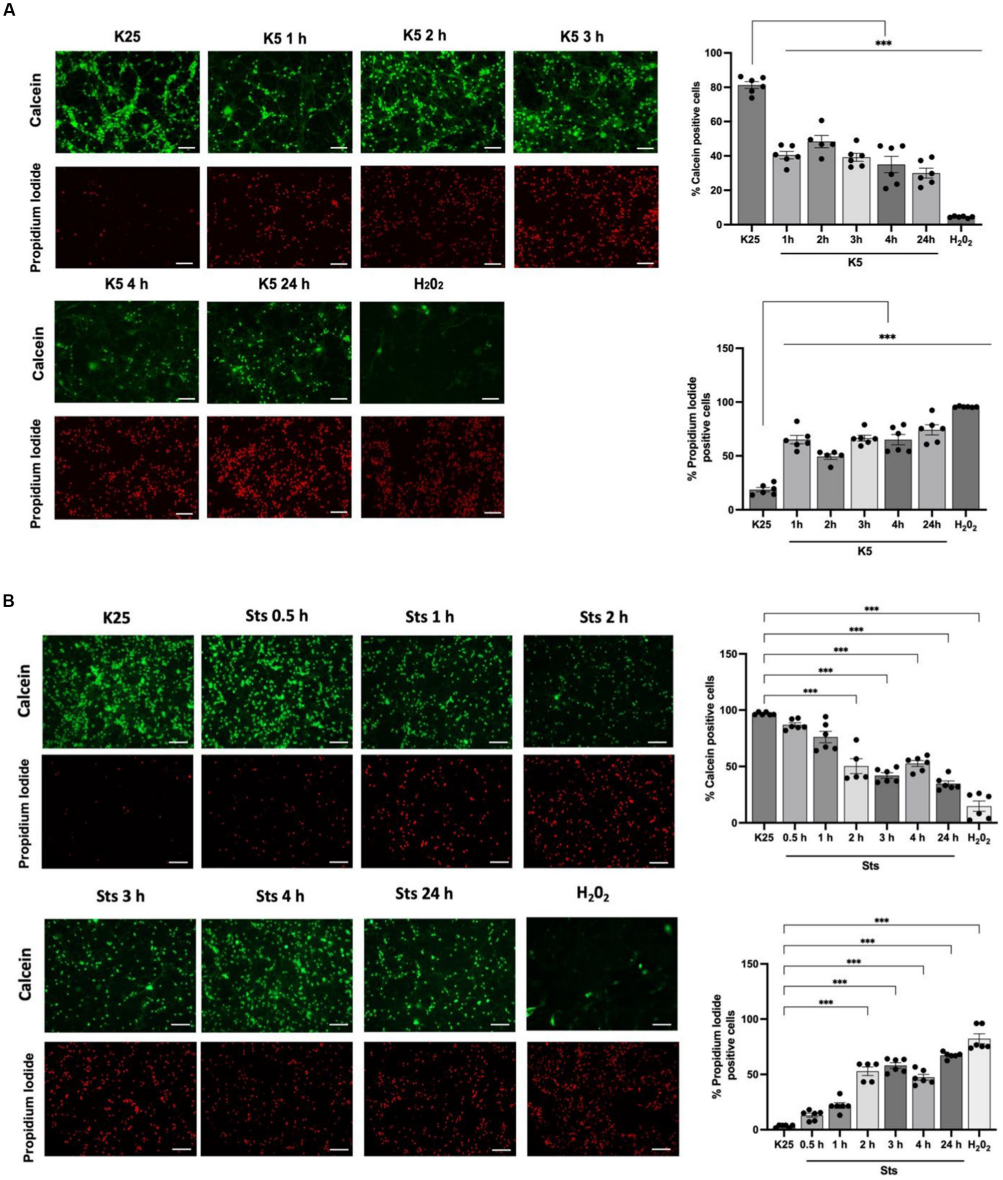
CGN are partially rescued when cells are returned to basal conditions after 1–24 h of K5 or Sts treatment. Seven DIV CGN were switched to a low potassium medium (5 mM KCl, K5) **(A)** or treated with Sts (0.5 μM) **(B)** for 1 to 5 h or with 100 μM H_2_0_2_ for 1 h and then cells were transferred to K25 medium, and neuronal viability was measured at 24 h by using calcein and propidium iodide (PI) dyes as detailed in Methods. The panel shows representative micrographs for each condition. Graphs show cell viability expressed as the percentage of calcein (living cells) or PI (dead cells) positive cells from total cells (calcein plus PI-positive cells). Bars represent the means ± SEM and the distribution of 3 independent experiments carried out by duplicate. ***p < 0.001. Data were analyzed with One way ANOVA followed by Dunnett’s multiple comparison test. Bars in the panels represent 100 μm.

CGN was treated with low potassium (5 mM) or staurosporine (0.5 μM) during 6–24 h and then neuronal viability was evaluated with calcein and propidium iodide as indicated in Methods. In line with previous studies (Valencia and Morán, 2001; Guemez-Gamboa and Morán, 2009; Ramiro-Cortés and Morán, 2009; Zaragoza-Campillo and Morán, 2017; Cid-Castro and Morán, 2021) we found a decrease in cell viability of 20% after 6 h of treatment with low potassium (K5) and by 60% after 24 h. In the case of staurosporine, we observed about 60% of cell death after 24 h of treatment. Therefore, the temporary window in which death is executed is from 18 to 24 h after the treatments (Figure 1). To know the minimal time required to activate the death process, we exposed CGN to K5 for short periods of time and then evaluated cell death after 24 h. When we evaluated calcein fluorescence we found that 1–3 h of treatment induce about 40–50% cell death 24 h later. However, when cells were treated for 5 h the cell death increased to 75–80%, which was similar to the cell death observed for continuous treatment during 24 h (Figure 2). Similar results were observed with Sts treated cultures; however, unlike K5, due to the chemical nature of Sts, it could be more difficult to thoroughly wash off the drug and prevent it from remaining longer in the preparation. When cells were treated with 100 μM H_2_0_2_ for 1 h, cell death was about 90% 24 h later.

### 3.2. Cell death conditions generate ROS

**Figure 3:**
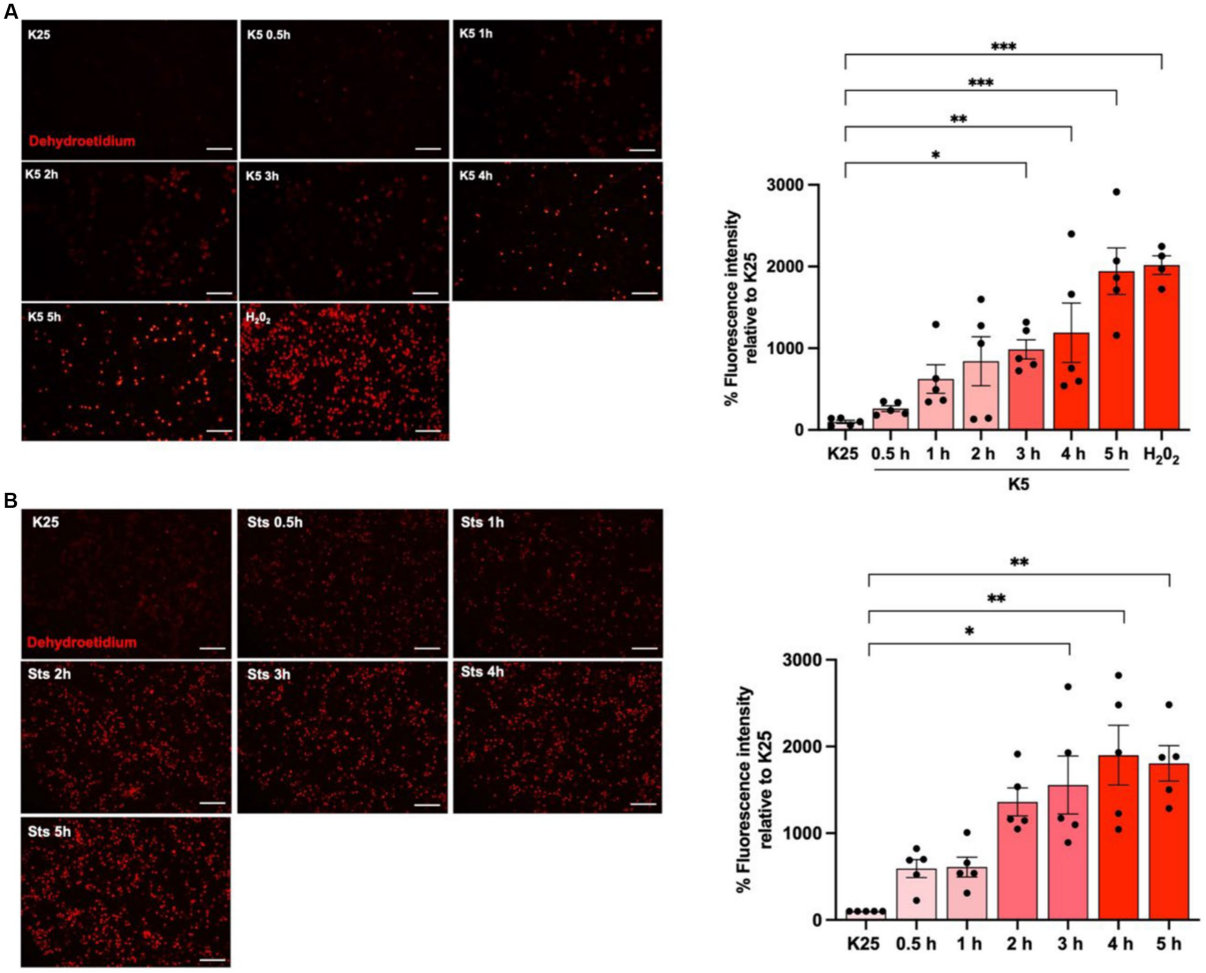
K5 and Sts induce reactive oxygen species generation. CGN grown in K25 medium were **(A)** transferred to a low potassium medium (K5) or **(B)** treated with staurosporine (0.5 mM, Sts) for 30 min to 5 h. Dihydroethidium was used to detect ROS as detailed in Methods. ROS levels are expressed as the percentage of fluorescence intensity of ethidium relative to K25. Treatment with H_2_0_2_ for 1 h (100 mM) was used as a positive control. Bars represent the means ± SEM and the distribution of 3 independent experiments carried out by duplicate. *p < 0.033, **p < 0.002, ***p < 0.001, significantly different from K25. Data were analyzed with One way ANOVA followed by Dunnett’s multiple comparison test. The bar in the panels represents 100 μm.

To evaluate ROS dynamics under cell death conditions, neuronal cultures were maintained in K25 medium for 7 DIV and then cells were switched to K5 medium or treated with staurosporine (0.5 μM) from 30 min to 5 h and ROS levels were measured with dihydroethidium (DHE) according to Material and methods. As previously reported (Valencia and Morán, 2001; Ramiro-Cortés and Morán, 2009; Zaragoza-Campillo and Morán, 2017), neurons treated with K5 showed a tendency to increase ROS levels from 30 min; however, we observed a statistically significant increase of 1,500% after 3 h that continued increasing after 5 h (Figure 3A). Similar results were observed for Sts. In this case, an increase of ROS production was also observed from 30 min, and after 3 h it was detected a significant increase of 900%, but the maximal ROS production was observed after 4 h (Figure 3B). We used 100 μM H_2_0_2_ for 1 h as a positive control and we observed that H_2_0_2_ increased the DHE signal by 2000% (Figure 3A). These data indicate that ROS are produced early, several hours (15 h) before neuronal death (Figure 3).

### 3.3. Death conditions and H_2_0_2_ induce *Txnip* expression

**Figure 4:**
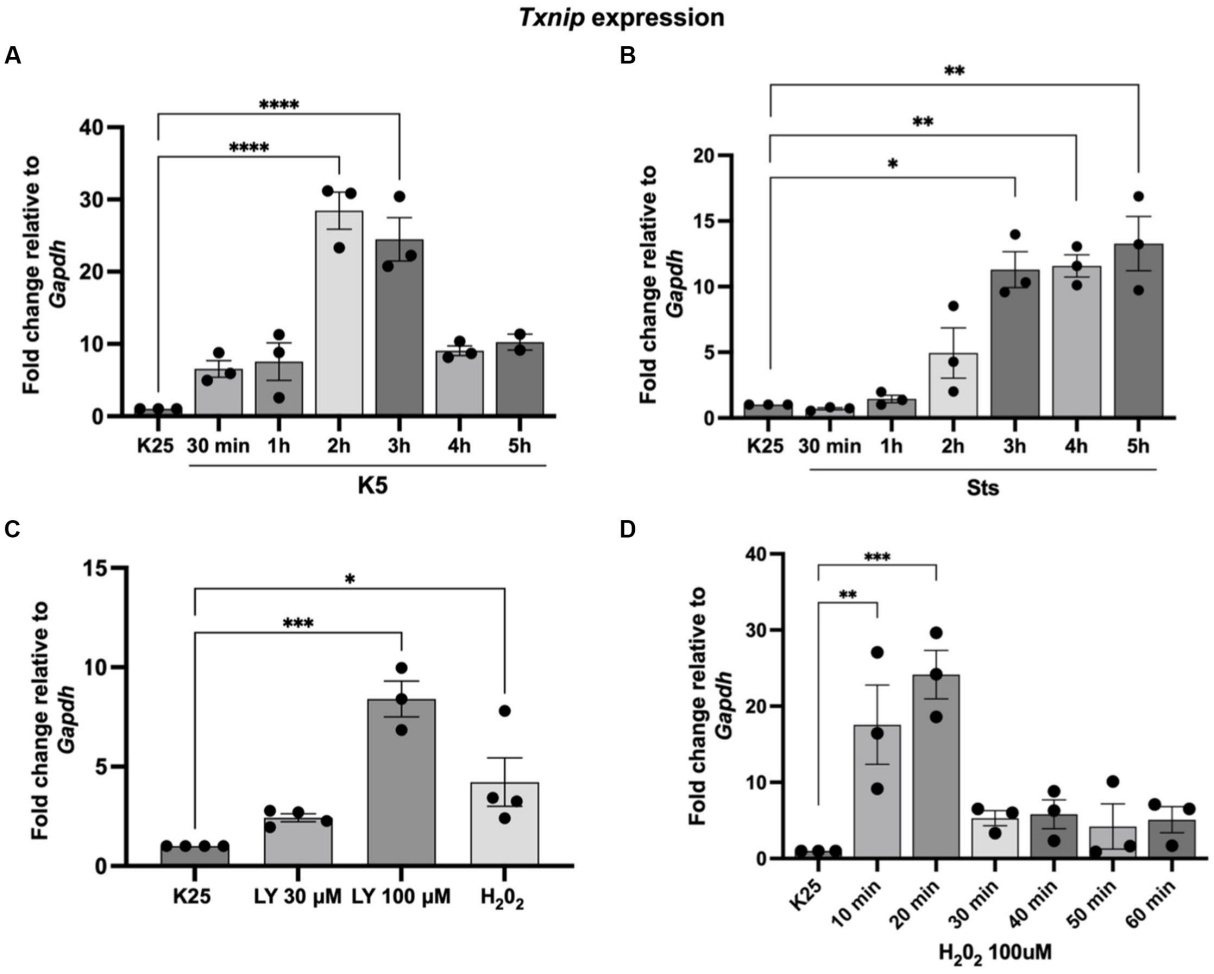
*Txnip* expression is induced by K5, Sts, H_2_0_2,_ and Akt inhibition. **(A)** Seven DIV CGN were cultured in K25 medium and switched to **(A)** K5 for 30 min to 5 h or **(B)** treated with Sts (0.5 mM) for 30 min) to 5 h or **(C)** LY290004 (30 mM and 100 mM) for 4 h or H_2_0_2_ 100 mM for 1 h **(D)** H_2_0_2_ (100 mM) for 10–60 min. After these times, RT-qPCR was performed as detailed in Methods. Txnip mRNA levels are expressed as fold change relative to *Gapdh*. Bars represent the means and the distribution of independent experiments. *p < 0.033, **p < 0.002, ***p < 0.001. Data were analyzed with One way ANOVA followed by Dunnett’s multiple comparison test compared to the K25 condition.

Our group previously found that TXNIP protein expression was induced by K5 and Sts (Zaragoza-Campillo and Morán, 2017). Furthermore, in that study, we demonstrated that TXNIP was also upregulated by ROS and that the antioxidant treatment decreased TXNIP levels induced by K5 and Sts (Zaragoza-Campillo and Morán, 2017). Here, we found that *Txnip* mRNA levels are also regulated by K5, Sts, and H_2_0_2_ (Figure 4). Cells were treated with K5 or Sts for 30 min to 5 h or with 100 μM H_2_0_2_ for 1 h. For K5, we found that *Txnip* levels showed a maximal increase after 2 h (about 20-fold) that decreased at 3 h, reaching an 8-fold increase after 4 and 5 h (Figure 4A).

When cells were treated with Sts, *Txnip* levels increased after 3 h (about 12-fold) and remained without change after 4 and 5 h of treatment (Figure 4B). To explore a possible involvement of Akt in the expression of *Txnip* we tested the effect of LY294002, an inhibitor of the IP3/Akt pathway. The inhibitor showed a dose-dependent increase of the *Txnip* levels (about 2 to 8-fold) after 4 h of treatment, supporting the idea that Akt is part of the signaling pathway (Figure 4C). Finally, we found that Txnip is 4-fold upregulated after 1 h of H_2_0_2_ treatment. To determine if there is an earlier response to the H_2_0_2_ we performed a temporal curve with H_2_0_2_ from 10 to 60 min. Under these conditions, *Txnip* levels showed a 17-fold increase after the first 10 min and a 24-fold increase at 20 min and after this time *Txnip* levels remained without change (4 to 5-fold increase) (Figure 4D). These data confirm that *Txnip* regulation is dependent on oxidant conditions and Akt inactivation.

### 3.4. FOXO 3 interacts with the *Txnip* promoter

**Figure 5:**
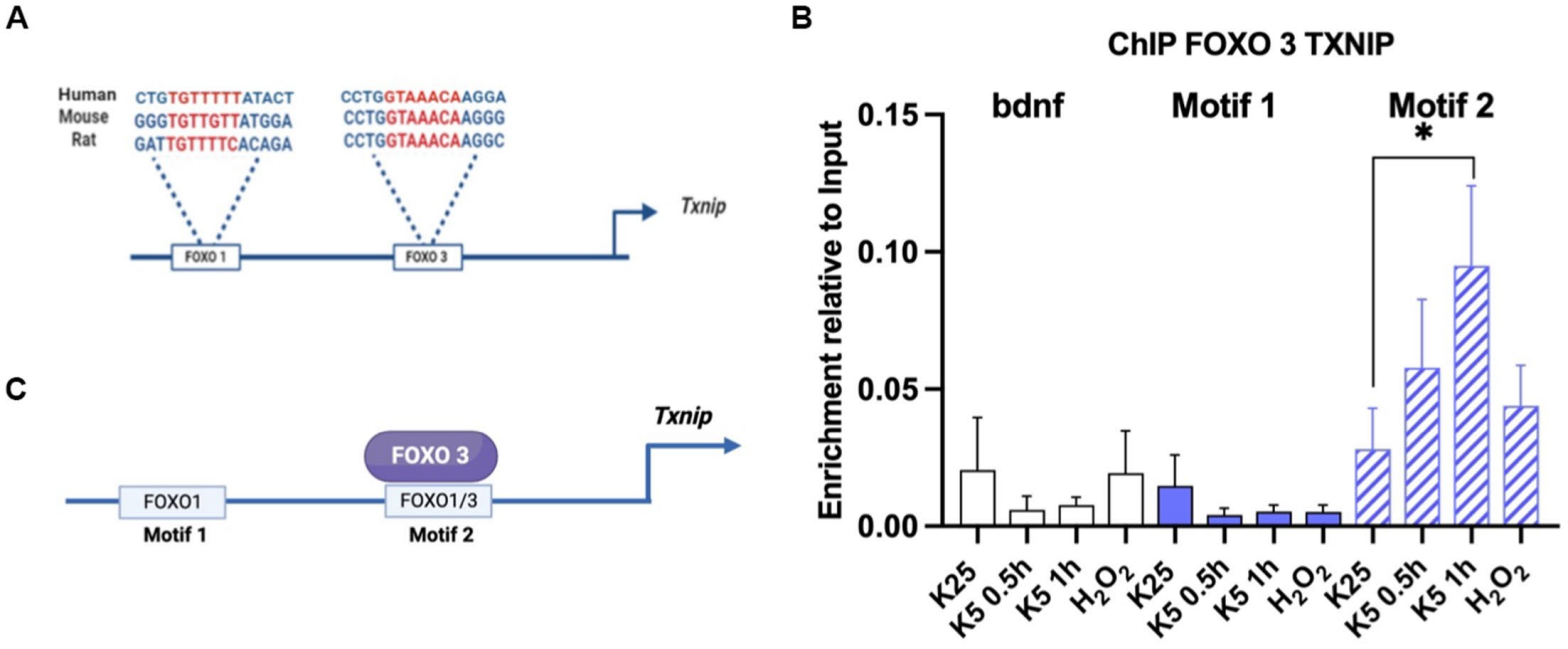
FOXO3 interacts with motif 2 in Txnip promoter. **(A)** FOXO 3 and FOXO 1 motifs in *Txnip* promoter are conserved in humans, mice, and rats. Promoter analysis was made using NCBI sequences and JASPAR-reported motifs for FOXO 1 and FOXO3 as described in Materials and Methods. **(B)** FOXO3 interacts with *Txnip* promoter after 1 h of K5 treatment. ChIP assay was performed by using mice CGN. Data are expressed as enrichment relative to the input. *Bdnf* primers were used as a control. Motif 1 and Motif 2 specific primers were designed for this technique. Bars represent the means ± SE of 2 independent assays. *p < 0.033. Data were analyzed with TWO ways ANOVA followed by Tukey’s multiple comparison test. **(C)** Graphical summary of the ChIP experiment. FOXO3 interacts with motif 2 in the *Txnip* promoter.

As mentioned before, Akt inactivation induces *Txnip* mRNA expression (Figure 4). It is also known that *Txnip* promoters share common motifs for different transcription factors, including FOXO (Wang et al., 2006; Baker et al., 2008; Tsubaki et al., 2020), whose activity seems to depend on the inactivation of Akt. To test a direct regulation of *Txnip* gene by FOXO, we performed a Chromatin immunoprecipitation (ChIP) assay for FOXO1 and FOXO3 in mice CGN cultures as detailed in Methods. We analyzed the promoter sequences in humans and rats (NCBI accession number NM_006472 and NM_001008767), and we found that these motifs are conserved across these species (Figure 5A). Neurons were switched to K5 medium or treated with 100 μM H_2_0_2_ for 1 h. Amplification regions were designed for the two putative FOXO binding sites. The ChIP experiment revealed that endogenous FOXO3 associates with the *Txnip* promoter in motif 2 after 1 h of K5 treatment (Figures 5B,C). We used primers for *Bdnf* motifs and antibodies against TFII as a control. The untreated neurons showed a small enrichment in comparison with K5 after 1 h. We have not found conclusive data for FOXO 1 (data not shown). Interestingly, the enrichment levels with the treatment of 100 μM H_2_0_2_ for 1 h did not show a significant difference with K25 (control; Figure 5C). Since the TXNIP protein is increased by the same treatment with H_2_0_2_ (Zaragoza-Campillo and Morán, 2017), ChIP data suggest that the gene is induced earlier and is a transient response.

### 3.5. FOXO 3 switches to the nucleus in response to death conditions and H_2_0_2_

**Figure 6:**
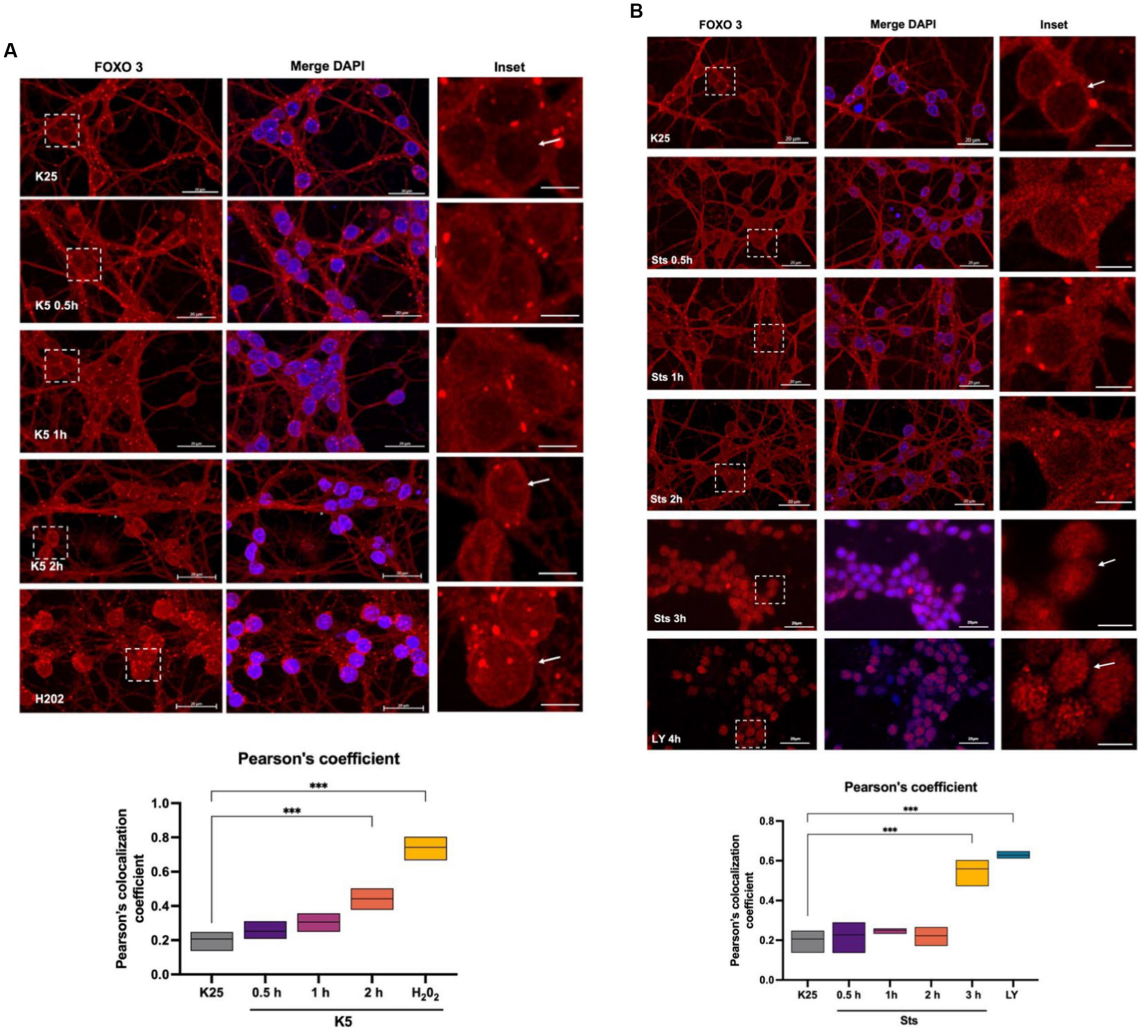
FOXO 3 translocates to the nucleus in response to K5, Sts. Representative micrographs of FOXO3 immunofluorescence of CGN treated with **(A)** K5 medium for 30 min, 1 h, or 2 h or **(B)** Sts (0.5 μM) for 30 min, 1 h, or 2 h. Immunofluorescence technique is described in Material and Methods. Alexa 488 secondary antibody (red) was used, and nuclei were stained with DAPI (blue). The right columns show the FOXO3 signal, the central panels show FOXO3 and DAPI merge signal and the left column is the dotted inset shown in the left panel. H_2_0_2_ (100 μM, 1 h) and LY294002 (100 μM, 4 h) were used as positive controls. Bars in the right and central panels represent 20 μm; bars in the left panels represent 5 μm. Pearson’s colocalization coefficients were determined in 3 independent experiments for each condition. Data were analyzed by one-way ANOVA followed by Dunnett’s test line in the floating bars representing the mean of the data. ***p < 0.001.

To identify FOXO3 localization during the death conditions and considering the *Txnip* expression data obtained (Figure 4), we performed a FOXO3 immunofluorescence of CGN treated with K5 and Sts during 30-, 60-, 120-, or 180-min. Figure 6A shows that under control conditions (K25) FOXO3 is mainly distributed in the cytoplasm. This is more evident in the inset where nucleus amplification is shown (Figure 6A). Pearson’s colocalization coefficient in this condition is 0.2 (Figure 6A graph). When cells were switched to a K5 medium, the FOXO3 signal increased in the nucleus, being evident after 2 h of K5 treatment (Figure 6A, arrows) with a colocalization coefficient of 0.4 (Figure 6A graph). The localization of FOXO3 in the nucleus after treatment with 100 μM H_2_0_2_ for 1 h showed a high Pearson’s coefficient (0.7) (Figure 6A, panel, graph). The translocation of FOXO3 correlates with the increase in ROS content.

When CGN was treated with staurosporine, cells showed an increase in FOXO3 nuclear localization after 3 h compared to K25. These data coincide with the expression of *Txnip* observed at 2 h with K5 and at 3 h with Sts (Figures 4A,B). To determine Akt participation in FOXO3 nuclear localization, we treated CGN with 100 μM LY294002 and we found a remarkable increase in FOXO3 nuclear distribution with a high Pearson’s coefficient (Figure 6B, arrow, and graph), which suggests that Akt inhibition regulates FOXO3 nuclear localization. Taking these results together we conclude that FOXO3 is a candidate to promote the Txnip expression in response to K5 and Sts in these cells.

### 3.6. TXNIP silencing decreases cell death

**Figure 7:**
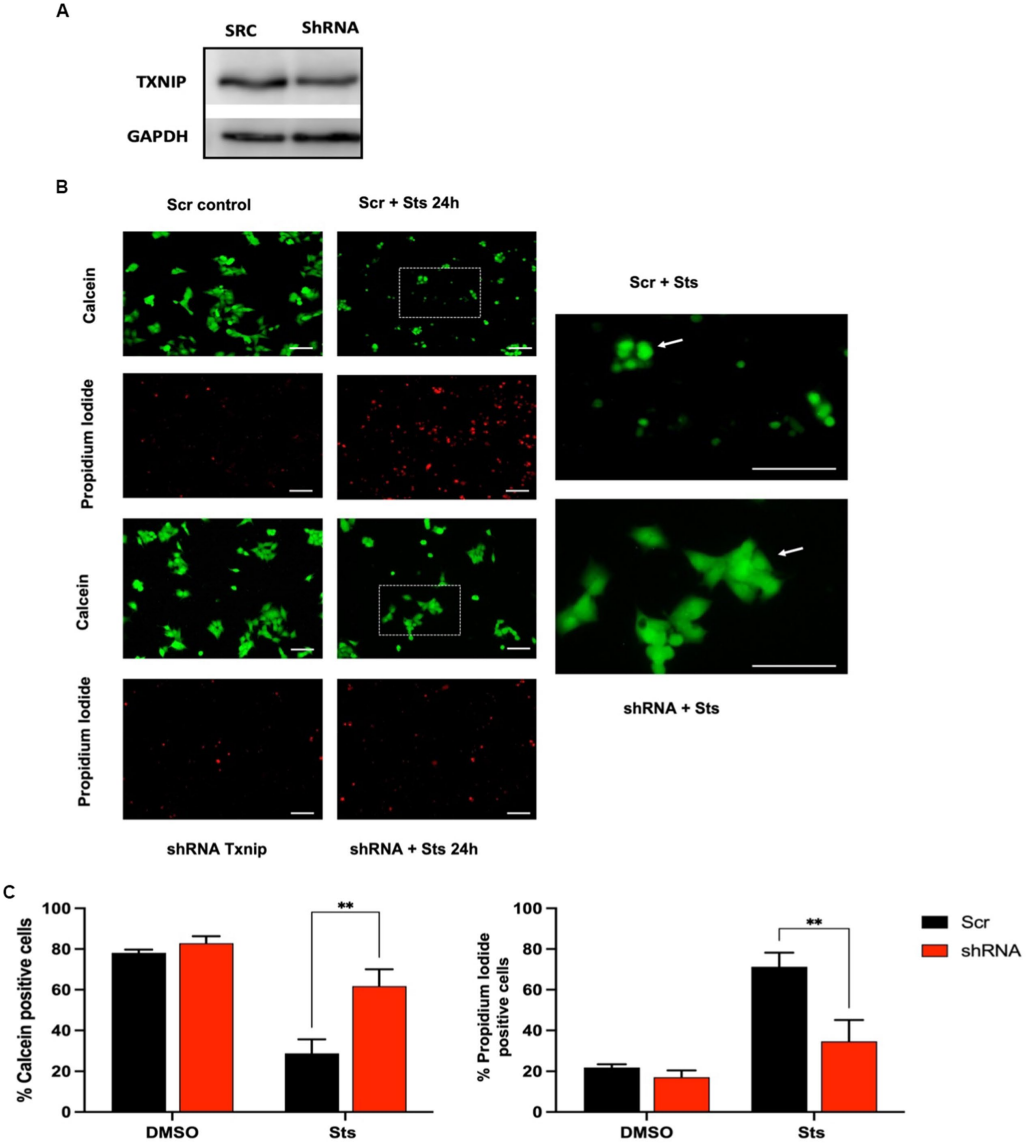
Txnip expression is required to induce cell death. Human neuroblastoma MSN cells were transfected with GFP-reporter scramble shRNA or Txnip shRNA with a transfection efficiency of 28%. After 2 DIV cells were treated with 1 μM Sts, cell viability was measured after 24 h with calcein/IP as indicated in Methods. **(A)** Representative TXNIP Western blot of cells transfected with Txnip shRNA for 24 h, GAPDH protein was used as control. **(B)** Representative micrographs of calcein/PI fluorescence of Scr or shRNA TXNIP transfected cells treated with Sts or vehicle (DMSO). The right panels show the magnification of the dotted insets shown in the central column. Bars in the right and central panels represent 50 μm; bars in the left panels represent 50 μm. **(C)** Quantification of calcein and PI-positive cells transfected with Scr or shRNA Txnip after 24 h of treatment with staurosporine or vehicle (DMSO). Graphs show cell viability expressed as the percentage of calcein (living cells) or PI (dead cells) positive cells from total cells (calcein plus PI-positive cells). Bars are the means ± SEM of 4 independent experiments. ***p < 0.002. Data were analyzed with two ways ANOVA followed by Sidak’s multiple comparison test.

We evaluated the participation of TXNIP in cell death in human MSN neuroblastoma cells treated with Sts. Potassium deprivation does not induce cell death in these cells. *Txnip* from humans and rats share 89% of identity and similar functions have been reported in both species. We found that MSN cells express *Txnip* under basal conditions and that shRNA decreases TXNIP protein levels by 10% 24 h after transfection (Figure 7A). MSN cells are less sensitive to staurosporine (data not shown), therefore we used 1 μM Sts to induce cell death. Control cells were treated with vehicle (DMSO) and transfection control was made by using an shRNA scramble sequence (Scr). Transfection efficiency was around 30% (data not shown) and no changes in the morphology and viability were found in control Scr or shRNA cells (Figure 7C).

When cells transfected with Scr were incubated for 24 h with Sts we observed a decrease of approximately 75% of cell viability measured as calcein staining (Figures 7A,C). In contrast, in cells transfected with shRNA against *Txnip*, Sts induced a decrease in cell viability of only 25%, which represents a 3-fold difference as compared with cells transfected with Scr sequence (Figures 7A,C). Data obtained with propidium iodide were in close agreement with calcein data (Figure 7C). Interestingly, Scr cells treated with Sts that survived after 24 h (stained with calcein) showed a rounder morphology compared to shRNA cells treated with Sts, which maintained the original morphology (Figure 7B amplification). We interpret this observation as an indication that the surviving Scr cells treated with Sts have already activated the apoptotic death pathway and therefore they will die in the following hours. These results demonstrate that TXNIP is required for cell death and that the lack of this protein increases neuronal viability under death conditions.

### 3.7. Neuronal death is dependent on JNK activation

**Figure 8:**
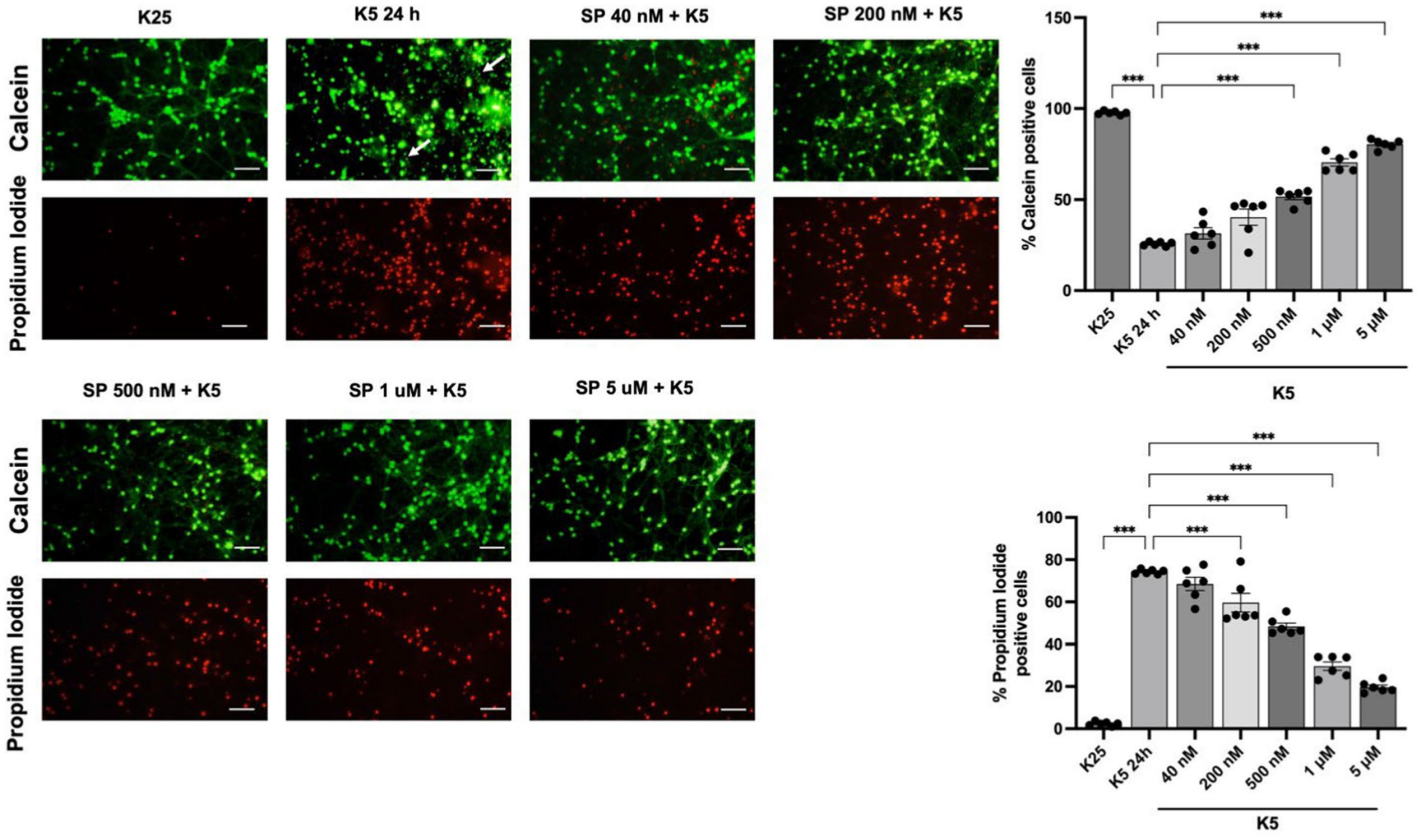
JNK pathway inhibition reduces neuronal death induced by K5. Seven DIV CGN cultured in K25 medium were switched to low potassium medium (K5) and treated with the JNK inhibitor SP600125 at a concentration ranging from 40 nM to 5 mM. Neuronal viability was measured after 24 h by using calcein and propidium iodide (PI) dyes as detailed in Methods. The panel shows representative micrographs for each condition. Graphs show cell viability expressed as the percentage of calcein (living cells) or PI (dead cells) positive cells from total cells (calcein plus PI-positive cells) Arrows in the K5 24h condition represent axonal degeneration. Bars represent the means and the distribution of independent experiments. Three independent experiments with two repetitions of each one were done. *p < 0.033, **p < 0.002, ***p < 0.001, significantly different from K5 24 h. Data were analyzed with One way ANOVA followed by Dunnett’s multiple comparison test. The bar in the panels represents 50 μm.

The role of JNK activation in apoptosis induced by K5 and Sts was confirmed in CGN by using SP600125, an inhibitor of the JNK protein in dose-dependent concentration (40 nM to 5 μM). Cell viability was measured 24 h after treatments. The reduction in the number of viable cells with K5 treatment was detected by calcein/propidium iodide technique. When cells were treated with K5 and the JNK inhibitor, CGN were markedly rescued from cell death. K5 treatment resulted in 26% of viable cells, in contrast to 80% of viable cells observed in the presence of SP (5 μM) (Figure 8). We also observed morphological changes associated with K5 treatment, including soma constriction and axonal degeneration, which were not evident in K5/SP treated cells (Figure 8, panel arrows).

## 4. Discussion

Apoptosis is a process characterized by an initial commitment phase, during which the cells become irreversibly destined to die (Belmokhtar et al., 2001). It has been suggested that, during development, a proportion of cerebellar granular neurons undergo physiological apoptosis caused by an absence of presynaptic glutamatergic inputs, which can be replicated *in vitro* by changing CGN from a depolarizing (25 mM KCl) to a hyperpolarized condition (low potassium level; Belmokhtar et al., 2001; Valencia and Morán, 2001; Contestabile, 2002; Guemez-Gamboa and Morán, 2009; Ramiro-Cortés and Morán, 2009; Zaragoza-Campillo and Morán, 2017; Cid-Castro and Morán, 2021). Apoptotic death of CGN can also be induced by staurosporine treatment (Saitoh et al., 2001; Valencia and Morán, 2001).

In the present study, we corroborated that about 50% of CGN die through K5 or Sts treatments. We also found that the commitment phase occurs at around 3–5 h, a time when the process is mainly irreversible, and cells can no longer be rescued from K5-induced death. In the case of Sts, we found similar results; however, due to the impossibility of completely removing Sts from cultures, it is possible that the cells are still exposed to Sts after washing and therefore the calculated times might not be as accurate. These data indicate that an early temporary window is required to activate the whole process of neuronal death and involves early ROS production (Valencia and Morán, 2001; Ramiro-Cortés and Morán, 2009; Cid-Castro and Morán, 2021) and MAPK signaling pathway activation, as previously reported (Ramiro-Cortés and Morán, 2009; Ramiro-Cortés et al., 2011). In the present study, we confirmed that JNK inhibition reduced cell death induced by K5. In a previous study, we observed that p38 inhibition markedly reduced Sts-induced death of CGN (Ramiro-Cortés et al., 2011).

Previous studies showed that when CGN are treated with antioxidants during the first 0–2 h, but not 5 h after K5 incubation, there is a prevention of the caspase activation and neuronal death measured after 24 h (Valencia and Morán, 2001). Here, we also detected a ROS increase after 30 min, but ROS production was statistically significant only after 3–5 h. We measured ROS by using dihydroethidium which detects cytoplasmic ROS. Recently, we detected a significant mitochondrial ROS production during the first 10-15 min of K5 and Sts treatment, which seems to be necessary for the progression of cell death because its inhibition by a mitochondrial antioxidant markedly prevented CGN death (Cid-Castro and Morán, 2021). We, therefore, believe that the mitochondrial ROS production after 10 min and cytosolic ROS produced 3–5 h later by NADPH-oxidase (Coyoy et al., 2008; Guemez-Gamboa and Morán, 2009) required to trigger an early molecular event necessary for cell death progression some hours later.

Zaragoza-Campillo and Morán (2017) reported that TXNIP protein increases after 2 and 3 h of K5 and Sts treatment, respectively. Interestingly, they also found TXNIP is induced by H_2_0_2_ and Akt inhibition and that antioxidants treatment prevents its expression, thus suggesting that this protein is regulated by ROS and Akt. Based on this evidence, we explored the *Txnip* transcriptional regulation by these two conditions in CGN and we first found that mRNA *Txnip* levels increased by 20 folds after 2 h of K5 treatment and approximately 12 folds after 3–5 h of Sts incubation. In line with previous results, we found that Akt inhibition for 4 h with LY294002 also induced a dose-dependent *Txnip* expression response. At 1 h treatment with H_2_0_2_ increased Txnip levels 4-fold. When we analyzed Txnip expression in a temporal curve from 10 min to 60 min with H_2_0, we found a rapid Txnip expression of 17-fold at 10 min and 24-fold at 20 min, and decreases to 5-fold from 30 min to 1 h. Akt deactivation by ROS has been described in several models, such as prostate cancer cells, cardiac cells, and N2A cells. This event is due to the specific cysteine oxidation and disulfide bond formation between Cys 297 and Cys 311 of the kinase domain, which induces its dephosphorylation and proteasomal degradation (Murata et al., 2003; Chetram et al., 2013; Ahmad et al., 2014).

The mechanism through which Akt inactivation leads to the upregulation of *Txnip* mRNA and protein has been described by Papadia et al. (2008) in cortical neurons. They reported an Akt-FOXO-TXNIP axe based on the evidence of the FOXO motifs found in the *Txnip* promoter, as well as the phosphorylation by Akt of FOXO transcription factors that leads to cytoplasmic recruitment by the 14–3-3 scaffold proteins that avoid their nuclear functions (Kloet and Burgering, 2011; Tzivion et al., 2011). This data is consistent with evidence reported by Webb et al. (2016) who found that FOXO 3 induced the expression of *Txnip* in neural progenitor cells NPCs by using a ChIP seq analysis. To explore whether Akt inactivation in our model promotes *Txnip* expression through FOXO transcription factors, we performed a ChIP assay in mice CGN for FOXO1 (data not shown) and FOXO3 for both motifs and we found that FOXO3 is positioned in motif 2 after 1 h of K5 treatment. This data suggests that in this model of cell death of CGN, FOXO3 is a strong candidate for inducing Txnip expression.

In agreement with the FOXO3 enrichment assays and *Txnip* mRNA expression, we observed that both K5 and Sts treatments induced FOXO3 redistribution to the nucleus after 2 h and 3 h of treatment, respectively. We also found a high FOXO3 nuclear localization induced by the Akt inhibitor LY294002 and H_2_0_2,_ reinforcing the evidence that ROS and Akt inactivation induce FOXO3 nuclear translocation. It is noteworthy that the enrichment levels with H_2_0_2_ treatment for 1 h were not statistically different from controls in the ChIP assays. In a time-curve of *Txnip* transcriptional regulation by H_2_0_2_, we found that up-regulation occurs in minutes (10–20), which coincides with the relatively high protein levels observed at 1 h by Zaragoza-Campillo and Morán. Interestingly, at this time (H_2_0_2_, 1 h) FOXO3 has a nuclear distribution even though it is no longer positioned in the *Txnip* promoter, probably due to other FOXO3 genes regulated during apoptosis.

The importance of *Txnip* expression in the death progression was demonstrated using human neuroblastoma MSN cells whose *Txnip* levels were reduced by using shRNA. Neuroblastoma cells do not respond to K5 because depolarizing conditions are not needed for their survival. For that reason, we only used staurosporine treatment as an apoptotic stimulus, which has been shown to be a death inductor in several neuroblastoma cell lines (López and Ferrer, 2000). In our model of neuroblastoma MSN cells, we found a basal TXNIP expression that is reduced by around 10% by the shRNA against *Txnip.* Probably this decrease contributes to a reduction in TXNIP levels in response to treatment with staurosporine. Although we do not know exactly how much the Txnip levels are reduced by shRNA under conditions of Sts treatment, we found that *Txnip* down expression improves cell viability 24 h after Sts treatment. We observed that 40% of cell death decreased in shRNA cells. It is possible that the observed *Txnip* decrease markedly reduces cell death induced by Sts because it prevents the *de novo* expression of Txnip in the presence of the pro-apoptotic stimulus, as occurs in SH-SY5Y cells (Pan et al., 2022). This result shows for the first time that TXNIP might play a critical role in the initial phase of cell death.

**Figure 9:**
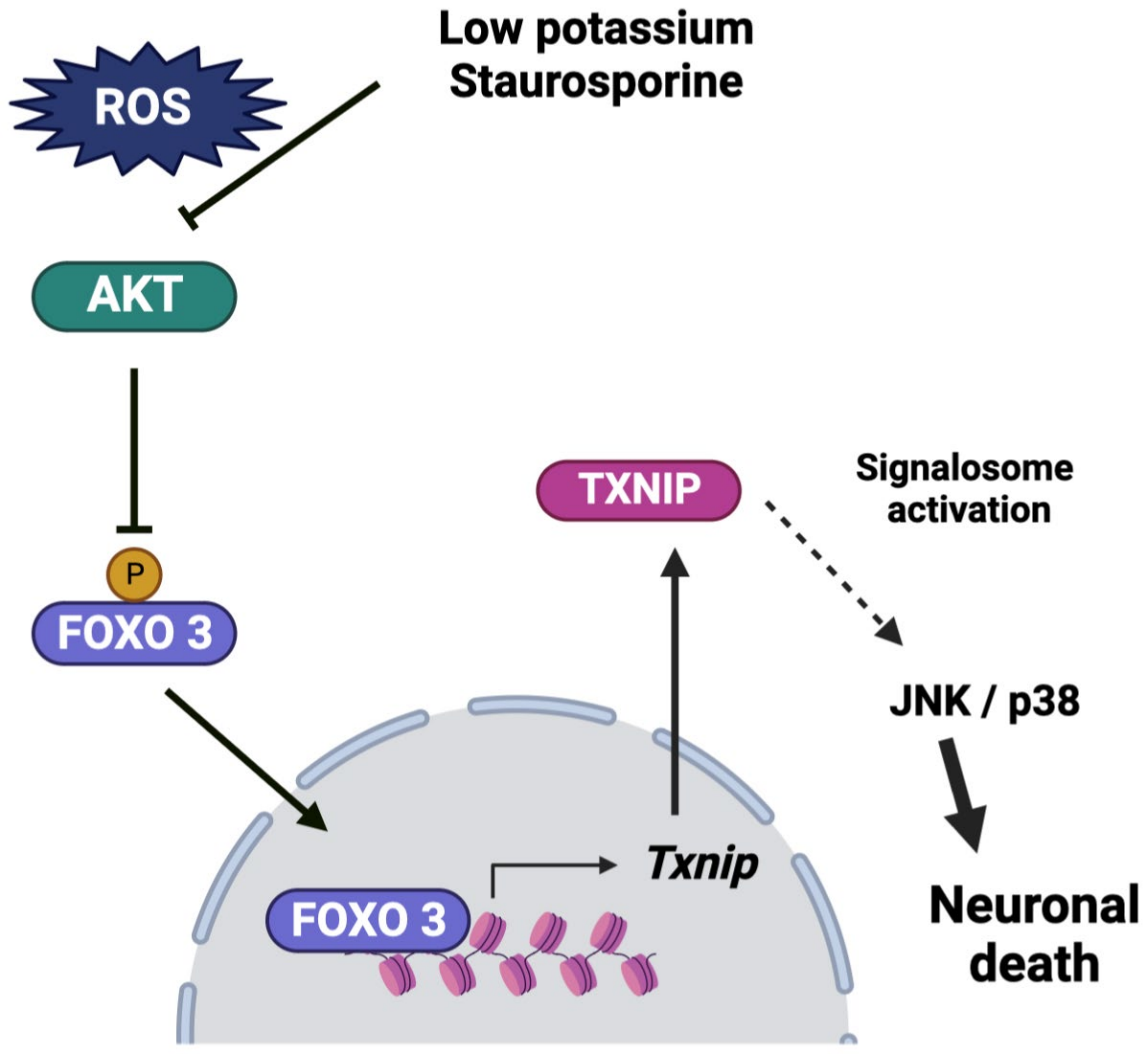
Schematic representation of the effect of low potassium and staurosporine on the Txnip expression and the progression of death. Low potassium and staurosporine share a common pathway to induce apoptotic death. The pathway is characterized by an early ROS production, which induces Akt inactivation by oxidation. This condition induces a reduction in the phosphorylation of FOXO3, which leads to its translocation to the nucleus, where it interacts directly with the Txnip promoter, probably inducing the expression of *Txnip.* The TXNIP produced may play a key role in the progression of death by activating the signalosome, which induces the activation of the JNK/p38 pathway and ultimately leads to cell death. Bio render program was used for the schema.

In conclusion, these results suggest that *Txnip* participates in cerebellar granule neuron death at early times in the cell death process and that TXNIP expression is dependent on reactive oxygen species production. We observed that ROS induces the inactivation of Akt, leading to the translocation of FOXO3 to the nucleus, where it interacts with the *Txnip* promoter at “Motif 2,” suggesting that FOXO3 is a candidate for TXNIP transcription under these conditions. The observed increase of TXNIP induced by ROS could contribute to the recruitment of Trx, which would activate ASK1 signaling, leading to the activation of the JNK/p38 pathway (Figure 9) and neuronal death.

## Data availability statement

The raw data supporting the conclusions of this article will be made available by the authors, without undue reservation.

## Ethics statement

This study was carried out following the accepted standards of animal care and with the procedures approved by the local Animal Care and Use Committee of the Instituto de Fisiología Celular, Universidad Nacional Autónoma de México (protocol number JMA175-21). The protocol used followed the Guidelines for the Care and Use of Mammals in Neuroscience as well as guidelines released by the Mexican Institutes of Health Research and the National Institutes of Health Guide for the care and use of laboratory animals (NIH Publication no. 8023, revised 1978). All efforts were made to minimize animal suffering and to reduce the number of animals used.

## Author contributions

BG-H carried out the experiments, contributed to analyzing and interpreting the data, drafted the manuscript, and participated in the design of the study. JM conceived the study and participated in its design and coordination and contributed to drafting the manuscript. All authors contributed to the article and approved the submitted version.

## Funding

This work was supported by the Consejo Nacional de Humanidades, Ciencias y Tecnologías (CONAHCYT) grant number 285184 and by the Dirección General de Asuntos del Personal Académico (DGAPA-PAPIIT, UNAM) grants number IN212019 and IN216422. Brenda García-Hernández conducted this study to fulfill the requirements of Programa de Doctorado en Ciencias Biomédicas of Universidad Nacional Autónoma de México, and received a doctoral scholarship from Consejo Nacional de Humanidades, Ciencias y Tecnologías (463895; CVU 755084).

## Conflict of interest

The authors declare that the research was conducted in the absence of any commercial or financial relationships that could be construed as a potential conflict of interest.

## Publisher’s note

All claims expressed in this article are solely those of the authors and do not necessarily represent those of their affiliated organizations, or those of the publisher, the editors and the reviewers. Any product that may be evaluated in this article, or claim that may be made by its manufacturer, is not guaranteed or endorsed by the publisher.

## Footnotes

^1^https://epd.epfl.ch/index.php
