# Corrigendum: *Txnip* expression promotes JNK-mediated neuronal death in response to reactive oxygen species

**DOI:** 10.3389/fnmol.2023.1275888

**Published:** 2023-09-12

**Authors:** Brenda García-Hernández, Julio Morán

**Affiliations:** División de Neurociencias, Instituto de Fisiología Celular, Universidad Nacional Autónoma de México, Mexico City, Mexico

**Keywords:** Thioredoxin Interacting Protein, FOXO3, reactive oxygen species, apoptosis, cerebellar granule neurons, MAPK, Akt

In the published article, there was an error in the Funding statement. The name of one of the funding sources is incomplete (CONAHCYT) and the name of the Ph.D. program was omitted.

The statement was previously stated that: This work was supported by the Consejo Nacional de Ciencia y Tecnología (CONACYT) grant number 285184 and by the Dirección General de Asuntos del Personal Académico (DGAPA-PAPIIT, UNAM) grants number IN212019 and IN216422. Brenda García-Hernández was supported by a doctoral fellowship from CONACYT fellowship number 463895. This study is part of the requirements for the Ph.D. degree in Biomedical Sciences of Brenda Vianey García Hernández at Universidad Nacional Autónoma de México.

The correct Funding statement appears below.

This work was supported by the Consejo Nacional de Humanidades, Ciencias y Tecnologías (CONAHCYT) grant number 285184 and by the Dirección General de Asuntos del Personal Académico (DGAPA-PAPIIT, UNAM) grants number IN212019 and IN216422. Brenda García-Hernández conducted this study to fulfill the requirements of Programa de Doctorado en Ciencias Biomédicas of Universidad Nacional Autónoma de México, and received a doctoral scholarship from Consejo Nacional de Humanidades, Ciencias y Tecnologías (463895; CVU 755084).

The authors apologize for this error and state that this does not change the scientific conclusions of the article in any way. The original article has been updated.

